# Severe asthma and the omalizumab option

**DOI:** 10.1186/1476-7961-6-4

**Published:** 2008-05-20

**Authors:** Christopher WT Miller, Narayanaswamy Krishnaswamy, Chambless Johnston, Guha Krishnaswamy

**Affiliations:** 1 Department of Medicine, Quillen College of Medicine, Johnson City, TN, USA; 2 Division of Allergy and Clinical Immunology, Quillen College of Medicine, Johnson City, TN, USA

## Abstract

Atopic diseases and asthma are increasing at a remarkable rate on a global scale. It is now well recognized that asthma is a chronic inflammatory disease of the airways. The inflammatory process in many patients is driven by an immunoglobulin E (IgE)-dependent process. Mast cell activation and release of mediators, in response to allergen and IgE, results in a cascade response, culminating in B lymphocyte, T lymphocyte, eosinophil, fibroblast, smooth muscle cell and endothelial activation. This complex cellular interaction, release of cytokines, chemokines and growth factors and inflammatory remodeling of the airways leads to chronic asthma. A subset of patients develops severe airway disease which can be extremely morbid and even fatal. While many treatments are available for asthma, it is still a chronic and incurable disease, characterized by exacerbation, hospitalizations and associated adverse effects of medications. Omalizumab is a new option for chronic asthma that acts by binding to and inhibiting the effects of IgE, thereby interfering with one aspect of the asthma cascade reviewed earlier. This is a humanized monoclonal antibody against IgE that has been shown to have many beneficial effects in asthma. Use of omalizumab may be influenced by the cost of the medication and some reported adverse effects including the rare possibility of anaphylaxis. When used in selected cases and carefully, omalizumab provides a very important tool in disease management. It has been shown to have additional effects in urticaria, angioedema, latex allergy and food allergy, but the data is limited and the indications far from clear. In addition to decreasing exacerbations, it has a steroid sparing role and hence may decrease adverse effects in some patients on high-dose glucocorticoids. Studies have shown improvement in quality of life measures in asthma following the administration of omalizumab, but the effects on pulmonary function are surprisingly small, suggesting a disconnect between pulmonary function, exacerbations and quality of life. Anaphylaxis may occur rarely with this agent and appropriate precautions have been recommended by the Food and Drug Administration (FDA). As currently practiced and as suggested by the new asthma guidelines, this biological agent is indicated in moderate or severe persistent allergic asthma (steps 5 and 6).

## Introduction

**Figure 1:**
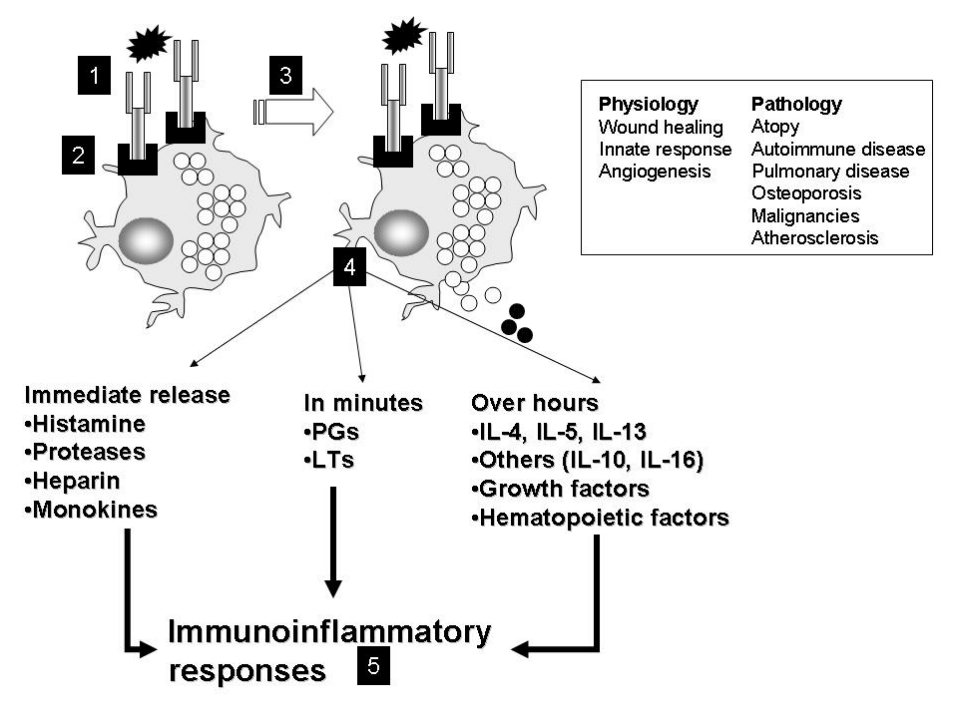
The allergen interacts with IgE on the surface of human mast cells, and  mediates signaling via the high affinity IgE receptor (FcεR1). FcεR1 aggregation is followed by  mast cell activation and degranulation.  Mast cells release a plethora of mediators which can then regulate eosinophil activation, Th2 skewing and B cell class switching to IgE. This sequence of events ultimatelyresults in  a number of immunologic and inflammatory responses.

**Figure 2:**
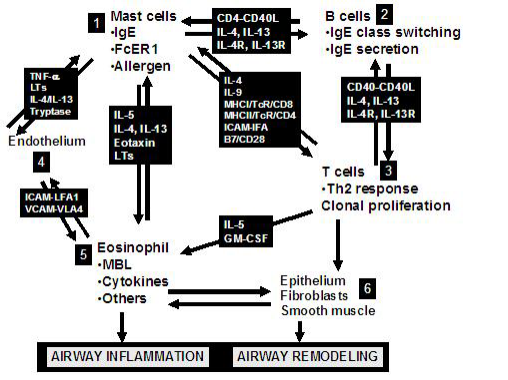
This figure demonstrates the pivotal position of IgE and mast cells in the initiation of the asthma inflammatory cascade. Mast cells  can interact with B cells  which can both interact with Th2 type T cells , an interaction mediated by cell surface cognate molecules and resulting in IgE class switching in B cells and cytokine expression. This can lead to endothelial activation  allowing the emigration of activated eosinophils  into airway tissue. Eosinophil-derived products (including major basic protein, cytokines, chemokines, and leukotrienes) can influence airway remodeling by inducing changes in airway cells  such as epithelium, fibroblasts and smooth muscles.

Asthma is a chronic inflammatory airway disease characterized by infiltration of the mucosa by inflammatory cells, mucus hypersecretion, subbasement membrane fibrosis, smooth muscle hypertrophy, epithelial loss and alterations of angiogenesis [1]. The result of these changes is airway obstruction, a cumulative effect of airway inflammation and remodeling changes. These various definitions are summarized in Table 1. Inflammatory processes that occur in asthma are summarized in Table 2. Several inflammatory events occur in asthma, resulting in the observed pathophysiological (Figure 1) and clinical effects [1-3]. Th2-type T cells secreting a distinctive set of cytokines [such as interleukin (IL)-4, IL-5 and IL-13] play a pivotal role in asthma. We and others have showed that IL-5 transcripts are detectable in the lungs of patients with asthma, dominantly derived from the T cell fraction [4,5]. IL-5 is pivotal to eosinophil activation and survival. The basic driving process in allergic asthma is the class switching to and secretion of IgE by B lymphocytes, in response to cytokines such as IL-4. This defines the atopic phenotype. IgE to environmental allergens (referred to as specific antibody) binds to the respective allergen with high affinity. The allergen interacts with IgE on the surface of human mast cells, and mediates signaling via the high affinity IgE receptor (FcεR1) (Figure 1). FcεR1 aggregation is followed by mast cell activation and degranulation. Mast cells release a plethora of mediators (Figures 1 and 2 and Table 1) which can, in turn, regulate eosinophil activation [6,7], Th2 skewing and B cell class switching to IgE [8,9]. Mast cells can also be activated by IgE-independent mechanisms such as bacterial infection [10], toll-like receptors, IL-1 [11] and by contact with either T cells [12] or fibroblasts [13]. IgE-mediated inflammatory responses may be responsible for a variety of atopic disorders, including rhinitis, asthma, eczema, food allergy, otitis media, anaphylaxis and asthma. Figure 2 demonstrates the pivotal position of IgE and mast cells in the initiation of the asthma inflammatory cascade. Mast cells (1) can interact with B cells (2) which can both interact with Th2 type T cells (3), an interaction mediated by cell surface cognate molecules and resulting in IgE class switching in B cells and cytokine expression. This can lead to endothelial activation (4), allowing the emigration of activated eosinophils (5) into airway tissue. Eosinophil-derived products (including major basic protein, cytokines, chemokines, and leukotrienes) can influence airway remodeling by inducing changes in airway cells (6) such as epithelium, fibroblasts and smooth muscles. Airway inflammation and airway remodeling together result in airway obstruction, which manifests clinically as dyspnea and wheezing.

**Table 1:** Asthma Definitions

**Manifestation**	**Definition**
**Asthma**	Inflammatory disease of the airways characterized by:
	• Infiltration by eosinophils, lymphocytes and neutrophils
	• Mast cell activation
	• Epithelial loss
	Associated reversible airway obstruction, recurrent symptoms and bronchial hyper-responsiveness
	Gene-by-environment interactions are important
**Inflammation**	Plays central role in asthma pathophysiology
	Mediated by T cells, lymphocytes, mast cells, eosinophils and epithelium. Other resident cells such as fibroblasts and smooth muscle play a role
**Airway remodeling**	As inflammation proceeds, other changes evolve leading to reparative or remodeling changes and include:
	• Sub-basement membrane thickening
	• Subepithelial fibrosis
	• Smooth muscle hypertrophy
	• Angiogenesis
	Mucus gland hyperplasia and hypersecretion
**Bronchoconstriction**	Airway narrowing resulting in wheezing
	Probably acutely due to release of mast cell mediators such as histamine, leukotrienes, and tryptase
	Obstruction could be mediated by multiple factors:
	• Smooth muscle contraction
	• Airway edema
	• Airway remodeling and fibrosis
**Bronchial Hyperresponsiveness**	Exaggerated bronchoconstrictive response to a wide variety of stimuli; best measured clinically by methacholine challenge testing; mediated by inflammation, remodeling and other airway changes that occur in asthma

**Table 2:** Inflammatory events in asthma: Role of Cytokine-IgE Axis and Inflammatory Cells

**Event**	**Cell types involved**	**Mediators**	**Cell type affected**	**Net effect**
**Inflammation**	**Antigen-presenting cell**	CSM	T cells, B cells	T cell activation Antibody synthesis
		TNF alpha	Endothelium	Cell adhesion, recruitment
	**Mast cells**	IL-4	T cells	Th2 cell polarization
		IL-4, IL-13	B cells	**IgE class switching**
		IL-13	Goblet cell	Mucus secretion CAM
		TNF-alpha	Endothelium	upregulation
		LTs	Smooth muscles	Contraction
		LTs, IL-5	Eosinophils	Chemoattraction, survival
		Histamine	Endothelium etc	Edema, bronchospasm
	**T cells**	IL-4, IL-13	B cells	**IgE class switching**
		IL-5	Eosinophils	Hematopoiesis, survival
		IL-9	Mast cells	BHR, mast cell growth
	**B cells**	**IgE**	Mast cells, basophil	Early phase response Mediator release
	**Eosinophil**	MBP	Epithelium, mast cells	Injury, histamine release
		LTs	Smooth muscle	Contraction
		Cytokines	Multiple types	Inflammation

CSM = Costimulatory molecule; LTs = leukotrienes; MBP = Major Basic Protein of Eosinophil; BHR = Bronchial hyperresponsiveness

The dominant mechanism of mast cell activation remains by IgE-antigen-FcεR1 interactions. Hence, inhibition of this pathway is likely to modulate an early phase of allergic inflammation. This concept led to the development of an anti-IgE antibody (omalizumab) as a treatment option for atopic diseases. The following sections will review the structure and clinical use of omalizumab.

## The problem

On the one hand, we are seeing an alarming increase in asthma incidence, severity and mortality. On the other, in spite of major advances in therapies, new delivery devices and improved potency of medications (inhaled steroids, long acting beta_2_-adrenergic agonists/LABA, combination medications, leukotriene antagonists), asthma is still a chronic and incurable disease. The major morbidity of asthma is associated with frequent exacerbations, emergency room visits, hospitalizations, and complications associated with some of the therapies. Moreover, a significant number of cases are poorly controlled despite combination therapy with high doses of inhaled corticosteroids and long-acting β-agonists, leukotriene antagonists and allergy immunotherapy. The GOAL study demonstrated that 38–53% of patients using "optimal therapy" continued to have poorly controlled disease [14]. This suggests a need for alternative strategies and agents. Such patients have additional comorbid problems such as esophageal reflux, chronic sinusitis or severe sensitivity to indoor inhalant allergens or pollutants, but continue to be symptomatic even when these factors are evaluated and treated. Moreover, the risk for dying in patients with severe asthma is fairly high, with a 6-fold increased risk for dying 3 years after hospital discharge. The new guidelines suggest that such patients with moderate or severe persistent asthma (step 5 or 6) may be candidates for the use of omalizumab.

## Omalizumab: historical and molecular aspects

**Figure 3:**
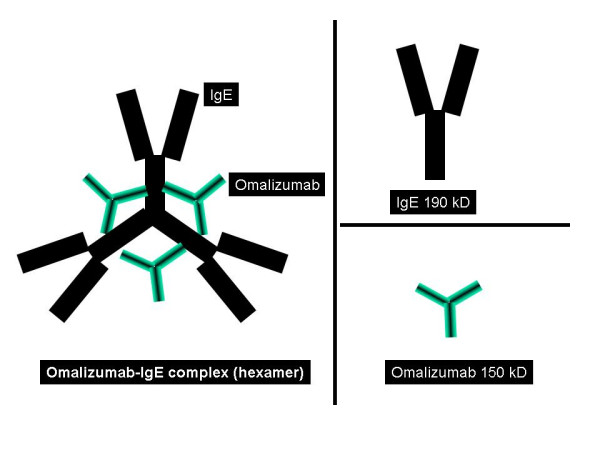
Omalizumab targets the Cε3 epitope on the fragment of IgE which binds the α chain of the high-affinity trimeric IgE receptor, thus blocking the binding of IgE with its receptor. On the left, formation of a hexamer complex of omalizumab-IgE is shown, and on the right molecular sizes of the IgE and Omalizumab molecules are shown.

The clinical applicability of anti-IgE products was conceptualized over 15 years ago [15-17], with a number of subsequent trials demonstrating their safety in several allergic conditions [18]. The idea for use in humans stemmed from the development of a murine monoclonal antibody termed MAE11, shown to block the interaction of IgE with basophils or mast cells without leading to cross-linking or degranulation. Multiple attempts were made to humanize this antibody and it was version 25 of these experiments which rendered a product with a profile similar to that of MAE11 [19]. This recombinant humanized monoclonal antibody was termed rhuMAb-E25, alternative names including IgE 025, omalizumab, and Xolair™ (which is how it is currently being marketed by Genentech/Novartis, South San Francisco, CA, and Tanox, Inc., Houston, TX). It consists of 95% human IgG_1 _and 5% murine IgG [20], a framework which reduced the potential for immunogenicity [16,21]. Omalizumab targets the Cε3 epitope on the fragment of IgE which binds the α chain of the high-affinity trimericthe IgE receptor (Figure 3), thus blocking the binding of IgE with its receptor [22].

## Omalizumab pharmacokinetics

Omalizumab is usually administered via the subcutaneous route, after which it will have a bioavailability of 62% and reach its peak serum concentration within seven to eight days [23]. Omalizumab has a long half-life (19 to 22 days), in part due to the slow removal by the hepatic reticuloendothelial system conferred by the IgG_1 _portion [24]. Availability of IgE and the receptor to which it is bound (low- or high-affinity) dictate the clinical effects of anti-IgE therapy, explaining a potential delay of weeks before any appreciable activity is noted [25]. The efficacy of omalizumab does not appear to differ when given subcutaneously or intravenously [26], with both routes causing a dramatic decrease in IgE levels. In spite of this, there may be more to explaining the mechanisms of omalizumab, as very few IgE molecules on a mast cell or basophil are required to cause sensitization [27]. With regards to other potential forms of administering the product, the study by Fahy et al. utilizing aerosolized E25 in allergic patients failed to demonstrate a significant decrease in serum IgE levels or any appreciable advantage over placebo in ameliorating early phase response, despite reporting the presence of anti-IgE in blood [28]. In addition, it appears this route is more immunogenic, as IgG and IgA antibodies to E25 were detected in one patient.

## Molecular structure and interactions with IgE

Omalizumab acts by selectively binding free IgE molecules (Figure 3), consequently disabling their interaction with the heavy chain FcεRI [29]. With IgE-mediated antigen cross-linking being inhibited, there will be no activation of mast cells or basophils [30]. This mechanism is made possible by the spatial arrangement of the Fc of IgE, composed of two heavy chains with binding sites for FcεRI, FcεRII (CD23), and anti-IgE symmetrically positioned along a central rotational axis through its two CH3 domains [31]. These three binding sites happen to be situated in an overlapping manner, hence occupation of one of these sites precludes any of the other two from additional interactions due to conformational changes [32,33]. Also, when there is binding of FcεRI to one CH3 domain, this inhibits anti-IgE from interacting with its antigenic site on the other CH3 domain. Interestingly, there may be attachment of anti-IgE to both CH3 domains, probably because this interaction does not lead to a significant change in conformation [31].

The affinity of anti-IgE for IgE is about 10^-10 ^M, which is comparable to that between FcεRI and IgE [31,34]. Omalizumab leads to a reversible decrease in the unbound serum IgE of 84–99% within approximately 1 hour, with low levels lasting 4–6 weeks after a single subcutaneous injection [23,35]. This is extremely significant, as expression of FcεRI is directly influenced by IgE levels, with the use of omalizumab down-regulating this receptor on the surface of basophils, dendritic cells (types 1 and 2), and skin mast cells (at a slower rate) [21,36-38]. This effect on dendritic cells will consequently disturb normal IgE-facilitated presentation of antigens to T cells. The use of omalizumab will cause a progressive dissociation of IgE from basophils extending over 3–4 weeks [39]. As FcεRI becomes free from IgE on the surface of mast cells and basophils, the receptors will suffer engulfment and degradation, with a similar process occurring to the newly-formed FcεRI molecules which never interact with IgE [31]. These events result in desensitization of both mast cells and basophils, leading to a much higher antigenic requirement to trigger an atopic reaction [37,40].

As mentioned, omalizumab is nonimmunogenic thanks to a specific property inherent to the product, which is a lack of interaction with IgE molecules already complexed with FcεRI or FcεRII, avoiding histamine release and potential anaphylactic reactions [16,31]. Anti-IgE does however interact with mIgE (membrane-bound IgE) on the surface of B cells which express this molecule, with subsequent cell lysis or inhibition [31].

As the IgE molecules become free from FcεRI, they will be bound by anti-IgE molecules circulating close by, forming immune complexes (Figure 3) which will provide additional protection by attaching to new incoming allergens and impede their binding to other FcεRI molecules [18,31]. The omalizumab-IgE immune complexes have not been shown to cause tissue damage or to fix complement, mainly due to their small size (the molecular weight is less than 1,000 kDa) and to the fact that there is very little accumulation of the complexes (the main route of excretion is urinary), even in the face of very high levels of IgE [19,41]. There may be local accumulation of immune complexes in the extravascular space (such as the mucosal epithelial lining), as anti-IgE can cross capillaries but is not able to return once complexed with IgE, and may therefore contribute to local protection against allergens [31]. The level of total IgE is actually elevated during use of omalizumab (as this assay includes the IgE-omalizumab complexes, which are cleared at a slower rate than IgE), while in reality free IgE is decreased [42].

## Molecular and clinical effects

As shown in Table 3, use of omalizumab has been evaluated in several allergic conditions, including allergic rhinitis, asthma, Churg-Strauss syndrome, atopic eczema, urticaria, angioedema, latex allergy, and concurrently with allergy immunotherapy to try to blunt reactions. While approved by the Food and Drug Administration (FDA) for asthma, the other indications need to be studied further. These will be reviewed later.

**Table 3:** Conditions shown to respond to omalizumab/anti-IgE* therapy

**Condition**	**Reference**
**Allergic rhinitis**	[66,69,85-89]
**Allergic asthma:**	
• **Moderate-severe persistent disease**	[55,64,90-94]
• **Steroid-sparing effect**	[57,95,96]
**Churg-Strauss syndrome**	[97,98]
Peanut anaphylaxis*	[52,70,71,99,100]
**Uticaria**	[78]
**Angioedema**	[101]
**Immunotherapy reactions**	[102]
**Rubber latex allergy**	[79]

One of the main focus points of recent trials has been the use of omalizumab in asthma. Table 4 lists the molecular and clinical effects of omalizumab in asthma. Researchers have analyzed both direct and indirect effects of the biological agent. Direct effects include decreased free level of IgE and decreased FcεRI expression on mast cells and basophils. Indirect effects are probably mediated by effects of mast cell activation and IgE regulation. These include decreased tissue levels of eosinophils, mast cells, T cells and B cells [43]. Omalizumab has been postulated to have a mast cell "stabilizing" effect [29].

**Table 4:** Presumed mechanisms of action of Omalizumab and effects in asthma

**Immunological Effects**	
**Direct effects**	Binding to constant region of IgE
	Decreased free levels of IgE
	Decreased mast cell/basophil FcεR1 expression
**Indirect effects**	Decreased mucosal eosinophils
	Decreased sputum eosinophils
	Decreased tissue IgE+ mast cells
	Decreased tissue B and T lymphocytes
	Inhibition of early and late phase reactions
	Improved BHR/unchanged BHR
	Improved response to methacholine challenge
	Mast cell stabilization: inhibits degranulation

**Clinical Effects**	

**Effects on asthma**	Decreased exacerbations
	Improved peak flow
	Small improvement in FEV_1_
	Decreased rescue β_2_-agonist use
	Improved quality of life
	Decreased mean nocturnal clinical score
	Decreased total asthma clinical score
	Decreased hospitalizations
	Improved asthma control
	Steroid-sparing effect

By way of its inhibition of several key mediators outlined in Figures 1 and 2 (e.g. proinflammatory cytokines, growth factor, nitric oxide), omalizumab significantly affects both early and late phases of asthma [23,44], diminishing the frequency of exacerbations and the need for inhaled steroids [45,46]. The late phase response is dependent on the influx and activation of granulocytes, and omalizumab acts by decreasing the number of eosinophils in sputum, blood, and nasal mucosa, achieved through inhibition of IL-5 secretion by mast cells and basophils and by directly inducing apoptosis of eosinophils [25,47-50]. Another beneficial effect is the decrease in airway responsiveness to adenosine 5'-monophosphate (a marker of airway inflammation in allergic asthma) seen with omalizumab use [51]. It needs to be understood that the early and late phase responses are experimental constructs and efficacy in these responses may not necessarily represent improvement in clinical asthma, which is best determined by improvements in quality of life measures, lung function, peak expiratory flow rates, and hospitalization or medication use. Nevertheless, the early and late phase responses provide one measure of studying drug efficacy in airway inflammatory disease.

The clinical effects on asthma are summarized in Table 4 and include improved asthma scores, decreased exacerbations, decreased steroid use, improved peak flows, decreased hospitalizations, and improved asthma control [52,53]. Several studies have demonstrated that administration of omalizumab is associated with a decreased incidence of exacerbations in asthma. Selected studies and reviews are listed in Table 3. Milgrom and colleagues evaluated high- and low-dose omalizumab (rhuMAbE25) in a placebo-controlled study of patients with asthma requiring inhaled or oral corticosteroids [54]. After a 4-week run-in period, 317 patients were randomly assigned to receive either placebo or high-dose (5.8 μg/Kg body weight per nanogram of IgE) or low-dose omalizumab (2.5 μg/Kg per nanogram of IgE) administered intravenously. For the first 12 weeks of the study, subjects were allowed to continue their regular doses of corticosteroids, while in the subsequent 8 weeks, the doses of corticosteroids were tapered, in an attempt to discontinue therapy. The investigators demonstrated lower asthma symptom scores in the treatment groups. More subjects in the anti-IgE group were able to lower doses or completely come off corticosteroids [54]. The INNOVATE study [46] was a double-blind, multicenter, parallel-group study of patients with asthma in which patients were randomized to receive omalizumab or placebo for 28 weeks. At the end of the 28 weeks, patients receiving omalizumab had a 26% reduction in clinically significant exacerbations, 50% reduction in numbers of severe exacerbations and a 44% reduction in emergency room visits [46] compared to placebo. The omalizumab-treated patients also experienced less hospitalizations, improved asthma scores, and greater improvements in peak expiratory flows and pulmonary functions. Such effects on exacerbations were also observed in several other studies of severe or moderate-severe persistent asthma [55-65]. In a meta-analysis, Holgate [65] demonstrated that omalizumab administration in severe asthma halved the rate of exacerbations and improved quality of life parameters.

## Effects of omalizumab in non-asthma conditions

The use of omalizumab has been successful in patients with both perennial [66] and seasonal rhinitis [26,67], in addition to a demonstrable benefit in patients with both allergic rhinitis and asthma [68]. The documented advantages include an improvement in overall quality of life, a decrease in the use of rescue antihistamine therapy, and fewer nasal symptoms [69]. The one caveat with regards to seasonal rhinitis is that the efficacy of omalizumab has only been shown to occur with doses capable of suppressing IgE levels to < 25 ng/ml [26,35,67].

A trial conducted with another monoclonal anti-IgE (TNX-901) reported an increase in the threshold for peanut sensitivity, signifying a potentially protective effect against severe adverse reactions deriving from unintentional ingestion [70]. A phase II trial with similar characteristics was initiated with omalizumab but put to a halt owing to reports of serious reactions to peanut flour in some patients before they received omalizumab [71]. Irrespective of this setback, further studies are planned for the near future [72].

Omalizumab has been used with a positive outcome in several small studies of urticaria. In the skin, omalizumab has a predominant influence on the late phase response, mainly due to the more rapid uncoupling of IgE from its low-affinity CD23 receptors (directly involved in the late phase) when compared to the high-affinity FcεRI [73]. Many studies have highlighted the positive effect of omalizumab on atopic dermatitis, especially in patients with moderately elevated IgE levels [74-76], but at least one study has documented failure of this treatment (the patients had very high levels of IgE) [77]. Despite this, IgE levels did not factor in the efficacy of omalizumab in patients with chronic urticaria, with a positive outcome observed with levels ranging from low to very high [78]. In addition, other studies have demonstrated a positive effect in alleviating symptoms in patients with latex allergy and in the treatment of cold-induced urticaria [79,80].

## Omalizumab: adverse events and safety

**Figure 4:**
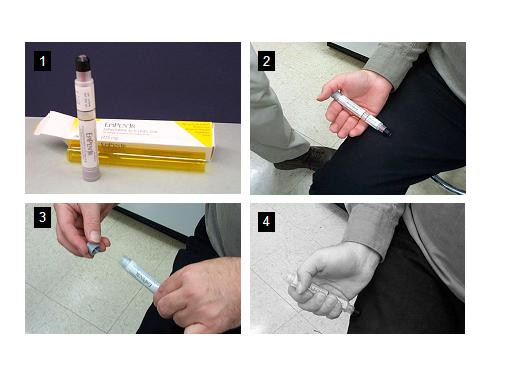
Administration of Epipen auto–injector. Panel 1 shows an auto–injector, and panels 2–4 demonstrate steps in arming the autoinjector and self-administration technique. The patient should grip the unit with the black tip pointing downward (panel 2) and proceed to pull off the gray safety release (panel 3). The injector should then be applied at a 90° angle with the outer thigh and held firmly for approximately 10 seconds after a click is heard (panel 4).

Tables 5 and 6 summarize the adverse effects seen to date with omalizumab. Of these, three events are probably important and need to be discussed. Local reactions occur fairly frequently in patients receiving omalizumab injections. These usually manifest as bruising, warmth, erythema, swelling, urticaria-like eruption. The local reactions are sometimes severe (in up to 12% injections). Anaphylaxis can occur in about 0.1% of injections. As reviewed by the American Academy Of Allergy, Asthma and Immunology and the American College of Allergy, Asthma and Immunology Joint Task Force Report [81], 35 patients had 41 episodes of anaphylaxis associated with omalizumab, corresponding to an anaphylaxis-reporting rate of 0.09% of patients. Of these 36 events for which time of reaction was known, 22 (61%) of the reactions occurred in the first 2 hours after injection, usually after one of the first three doses. The Task Force concluded that an observation time of 2 hours for the first three injections and 30 minutes after that would have captured 75% of the reactions [81]. That still leaves 25% of reactions that could pose a risk but the combination of anaphylaxis education, provision of an epinephrine auto–injector and close monitoring should be effective in averting or treating anaphylaxis, should that occur. Anaphylaxis management is discussed in Table 7 and administration of Epipen auto–injector is demonstrated in Figure 4. Finally, initial studies showed a small increase in the numbers of malignancies in the treated individuals. Out of 4127 omalizumab-treated patients, 20 cases of malignancy appeared. These included breast, prostate, melanoma, and parotid tumors. One case of lymphoma was recorded. Initial data suggested that these malignancies occurred in 0.5% of omalizumab-treated patients compared to 0.2% of controls. A later review of that data by an independent review group and comparisons with the SEER database of cancer incidence suggested that the incidence with omalizumab was no different from that of the general population. Currently under way is the EXCEL trial, a long-term prospective study which addresses the specific issue of the risk of cancer associated with omalizumab therapy.

**Table 5:** Adverse events with Omalizumab

**System**	**Reaction/AE**	**Frequency**	**Other aspects**
**Systemic**	Malignancy	20/4127 patients	0.5% versus 0.2% control Breast, prostate, melanoma Skin cancer, parotid, etc.
	Anaphylaxis	1/1000 patients	0.1%–0.2% 60% within 1–3^rd ^dose <2 hrs 14% after 4^th ^dose, <30 mins
	Viral infection	23%	Fever, myalgia, etc. 53% versus 42% control
	Parasitic infection	36/68	Odds risk 1.98 Geohelminths
	Immunogenicity	<0.1%	Antibody to Omalizumab
**Cutaneous**	Injection site reaction	45%	Warmth, erythema Bruising, burning
	Skin eruption*	6%	Dermatitis, urticaria
**Respiratory**	Infections*	20%	
**Sinus**	Sinusitis*	15%	
**CNS**	Headache*	15%	
**Pharynx**	Pharyngitis*	11%	
**Platelets**	Thrombocytopenia	?	Post-marketing observation
**Integument**	Alopecia	?	Post-marketing observation

* Reactions occurring at the same rate as placebo/controls

**Table 6:** Omalizumab-Other features

**Drug interactions**	Unknown
**Pregnancy**	Category B
**Nursing**	Excreted in milk*
**Fertility**	Study with monkeys shows no effect
**Pediatric use**	Safety below 12 years unknown
**Geriatric use**	Not enough data

* No human data available

**Table 7:** Anaphylaxis management

**Immediate treatment**	Assessment of breathing, circulation and orientation
	Inject epinephrine 0.3 mg intramuscularly in lateral thigh
	Activate emergency services (911)
	Patient to be placed in recumbent position
	Establish and maintain airway Oxygen
	Establish an intravenous line
	Use nebulized beta-agonist for bronchospasm +/- corticosteroids and antihistamines
	
**Long-term prevention**	Patient education
	Provision of epinephrine autoinjector
	Anaphylaxis identification (card, bracelet)
	Xolair information sheet

Interestingly, no cases of serum sickness or anti-omalizumab monoclonal antibodies have been recorded to date, although Dreyfuss and Randolph described one case of anaphylactoid reaction to omalizumab which evolved later into a serum sickness-like syndrome after pretreatment with a nonsteroidal agent [82]. This required discontinuation of the medication. Omalizumab is pregnancy category B and is excreted in milk (Table 6).

## Insurance and reimbursement issues

The use of omalizumab was first approved in the United States in 2003 and in Europe in 2005 [30]. In the U.S., Apart from certain particularities which may vary according to region, most issues (i.e. reimbursement, preauthorization, continued coverage) have been standardized [83]. Insurance companies may differ with regards to copayment and deductible specifications.

In order for a prescription of omalizumab to be filled, a participating specialty pharmacy will require a "statement of medical necessity" (SMN). This document will include the diagnosis of allergic asthma, a list of current medications, the patient's serum IgE level, and the positive results of the allergy testing performed. The pharmacy will then proceed to contact the patient's insurance company and determine if coverage is provided.

In the event of a claim denial, Genentech has set up a Single Point of Contact (SPOC) program which offers services such as benefit investigations and appeals assistance free of charge, apart from assisting patients in obtaining reimbursement for their products. If the patient does not have insurance or has been rejected for insurance and meets certain financial criteria, this program may provide medications free or at a reduced rate.

Even when approval for omalizumab use is given, it is usually only for a period of six months. Most insurance companies will then require a "recertification of continued use" to establish if the medication is truly providing any benefit. Documentation must be provided by the physician that there has been an improvement in symptoms or a reduced frequency of exacerbations, usually at six and twelve months after omalizumab has been started. The physician may also opt to document improvement via pulmonary function testing. Of note, the efficacy of omalizumab is determined after a minimum treatment period of twelve weeks, due to the delay in onset (as specified above).

## Indications for omalizumab

Some criteria for approval of use that have become virtually universal across insurance plans include: (1) a diagnosis of moderate to severe persistent asthma, (2) age ≥ 12 years, (3) serum IgE levels between 30 and 700 IU/ml, and (4) a positive skin test or blood test (such as radioallergosorbent test – RAST) for at least one perennial aeroallergen. The recent asthma guidelines [1] recommend the use of omalizumab for moderate to severe persistent asthma (steps 5 and 6) specifically.

Additional criteria, such as need for pre-bronchodilator FEV1 within the past six months, negative current smoking status, use of high-dose inhaled steroids for at least six weeks (usually in association with a long-acting bronchodilator and leukotriene modifier), and presence of active symptoms (i.e. daily use of bronchodilators/constant need for rescue therapy, asthma-related hospitalization within the past twelve months) have been variably used.

It is important to note that omalizumab will not be approved for treatment of asthma exacerbations (acute bronchospasm or status asthmaticus). Allergic rhinitis is not considered an indication for omalizumab use and will not usually be covered by insurance carriers. However, when used in patients with asthma and concurrent rhinitis, omalizumab is likely to be effective in controlling upper airway symptoms. Omalizumab is currently approved for use in adults and children over 12 years of age; studies are ongoing for children under the age of 12.

The acceptable codes for reimbursement (according to the Ninth Revision of the International Classification of Disease – ICD-9) are 493.00, 493.1, 493.9. The current procedural terminology (CPT) code for omalizumab use is 90772 and can be billed for each given injection. The CPT codes for percutaneous skin testing are 95024, 95028, 95004. In addition to the CPT code, there is a J code applicable for omalizumab, which is J2357, utilized when the medication is given in the office setting. The HCPC code for therapeutic, prophylactic, or diagnostic injections of omalizumab is 90772. HCPC codes describing chemotherapy injections are not to be used.

## Omalizumab dosing and duration of therapy

Omalizumab dose is calculated based on the patient's baseline serum IgE and the patient's body weight. The decision on the dose and frequency of administration can be made using the standardized tables (Tables 8 and 9). The dosage of omalizumab chosen is calculated to result in neutralization of free IgE to levels < 5% at baseline (0.016 mg/kg of omalizumab per IU/mL per 4 weeks). The total IgE may actually increase secondary to formation of omalizumab-IgE complexes and hence the IgE levels should not be measured after treatment initiation. The patient needs to understand that this is a long-term therapy, administered subcutaneously every 2 or 4 weeks depending on body weight and baseline IgE level [83]. Each vial of omalizumab contains 150 mg of the drug. After reconstitution with 1.4 mL of sterile water for injection, the vial will contain 150 mg of omalizumab in 1.2 mL of liquid. Swirling is necessary to accomplish complete solution of the medication, which is then administered subcutaneously. Two caveats about administration exist, namely that the total dose cannot exceed 375 mg and the dose/single injection should not exceed 150 mg, resulting in the need sometimes for multiple injections. Observation for anaphylactic events is required as discussed under the adverse events section. The cost per vial of omalizumab is $470, and depending on dosages, the cost/year may vary between $6000 and $36000 [83]. A recent study by Wu et al. assessed the cost-effectiveness of omalizumab in the treatment of asthmatic patients [84]. They found that the costs amount to $821,000 per quality-adjusted life year (QALY) and $120 for each symptom-free day gained. They go on to conclude that omalizumab therapy is only cost-effective in seriously ill asthmatics failing to achieve adequate control with other treatment modalities.

**Table 8:** Administration every 4 weeks

**Pre-treatment**	**Body weight (Kg)**			
	
**Serum IgE Level (IU/mL)**	**30–60**	**>60–70**	**>70–90**	**>90–150**
>30–100	150	150	150	300
>100–200	300	300	300	
>200–300	300			
>300–400*				
>400–500*				
>500–600*				

* See Table 8 for details on dosing

**Table 9:** Administration every 2 weeks

**Pre-treatment**	**Body weight (Kg)**			
	
**Serum IgE Level (IU/mL)**	**30–60**	**>60–70**	**>70–90**	**>90–150**
>30–100*				
>100–200*				225
>200–300*		225	225	300
>300–400	225	225	300	
>400–500	300	300	375	
>500–600	300	375	**DO NOT DOSE**	
>600–700	375	**DO NOT DOSE**		

* See Table 7 for further details on dosing.

## Conclusion

There are several unmet needs in asthma. Severe asthma is a difficult disease to control and is associated with significant morbidity and mortality. Omalizumab, a humanized monoclonal antibody, has been shown to be effective in difficult-to-control asthma. Further studies are required to determine which patients may most benefit from omalizumab.

## Authors' contributions

CWTM participated in the design of this review, discussed the molecular mechanisms of omalizumab along with insurance and reimbursement issues, apart from drafting the manuscript and its revisions, NK and CJ assisted with discussing the clinical and pharmacologic aspects of omalizumab, GK conceptualized the study, provided the framework and guided the entire editing process. All authors read and approved the final manuscript.
